# Primate abundance and habitat preferences on the lower Urubamba and Tambo rivers, central–eastern Peruvian Amazonia

**DOI:** 10.1007/s10329-013-0357-3

**Published:** 2013-05-10

**Authors:** Rolando Aquino, Fanny M. Cornejo, Eckhard W. Heymann

**Affiliations:** 1 Facultad de Ciencias Biológicas, Universidad Nacional Mayor de San Marcos, Lima, Apartado 575, Iquitos, Peru; 2 Museo de Historia Natural, Universidad Nacional Mayor de San Marcos, Lima, Av. Arenales 1256, Lima-14, Peru; 3 Abteilung Verhaltensökologie and Soziobiologie, Deutsches Primatenzentrum, Kellnerweg 4, 37077 Göttingen, Germany

**Keywords:** Diversity, Habitat, Bamboo forest, Primate communities, Geographic distribution, Population density

## Abstract

We report information on population density, group size, and habitat preferences of primates along the lower Río Urubamba and in the Río Urubamba–Río Tambo interfluvium, in central–eastern Peruvian Amazonia, an area that has been little explored with regard to its primate fauna. During 425 km of transect walks in October–November 2008 and April–May 2009 totally 174 groups of nine primate species were encountered, the most common being *Callicebus brunneus* (45 groups), *Saguinus imperator* (41 groups), and *Aotus nigriceps* (26 groups). Group sizes were smallest for *A. nigriceps* and *C. brunneus* (mean of 2.8 and 2.9, respectively) and largest for *Saimiri boliviensis* (mean 15.6). Population densities were lowest for *Lagothrix cana* (3.3 individuals/km^2^) and highest for *A. nigriceps* (31.1 individuals/km^2^). Groups of *C. brunneus*, *S. imperator*, *S. boliviensis*, *Cebus albifrons*, and *Cebus apella* were most frequently (83 % of sightings) encountered in semi-dense or in open primary forest that included stands of bamboo (*Guadua sarcocarpa*) or where bamboo was a very common species.

## Introduction

Western Amazonia, particularly south-eastern Peru, is among the Neotropical regions with the highest primate alpha diversity (Terborgh 1983; Peres and Janson 1999). In Peru, habitats harbouring this diversity are coming increasingly under threat, because of country-wide granting of concessions for exploitation of oil and timber (Perú Petro 2007). As recently shown by Kolowski and Alonso (2012), even the comparatively minor disturbance associated with seismic exploration (which generally precedes oil exploitation) results in a decrease in encounters with several primate species, particularly *Ateles belzebuth*. For many areas that will be exploited for oil or other resources in the future, either no data on their primate communities or only inventories are available, but baseline data on population densities that could be used to evaluate the effect of exploitation are lacking. Amongst these areas are the lower Río Urubamba and the interfluvium between this river and the Río Tambo. Biodiversity inventories on lower Río Urubamba indicate that it harbours one of the most species-rich mammal communities in the Neotropics (103 species of small mammals: Solari et al. 2001; 64 species of medium–large mammals, including potentially 14 primate species: Boddicker et al. 2001). The area is of biogeographical interest for several other reasons:

1. It is located between or close to the headwaters of several rivers, for example the Río Yurua and Río Purús, which may serve as geographic barriers for primates and other mammals (Hershkovitz 1977; Patton et al. 2000; Gascon et al. 2000), although this barrier function may break down at the headwaters (Peres et al. 1996);
2. Consequently, ranges of different species/subspecies may adjoin here as is observed for *Pithecia* and *Cebus* (Aquino and Encarnación 1994);
3. The Río Urubamba is believed to be the southern and western limit of the distribution of *Cacajao calvus ucayalii* in south-eastern Peru (Hershkovitz 1987); and
4. The presence in the Río Urubamba–Río Tambo area of forests with extensive stands of bamboo (*Guadua*) (Comiskey et al. 2001) provides the opportunity to compare primate communities in different types of habitat within a narrow region.

These considerations and the fact that the area will be subjected to oil exploitation in the future motivated us to conduct surveys on the lower Río Urubamba and in the Río Urubamba–Río Tambo interfluvium. During transect censuses we collected data to determine population densities, group size, and habitat preferences of the primates in this area.

**Fig. 1:**
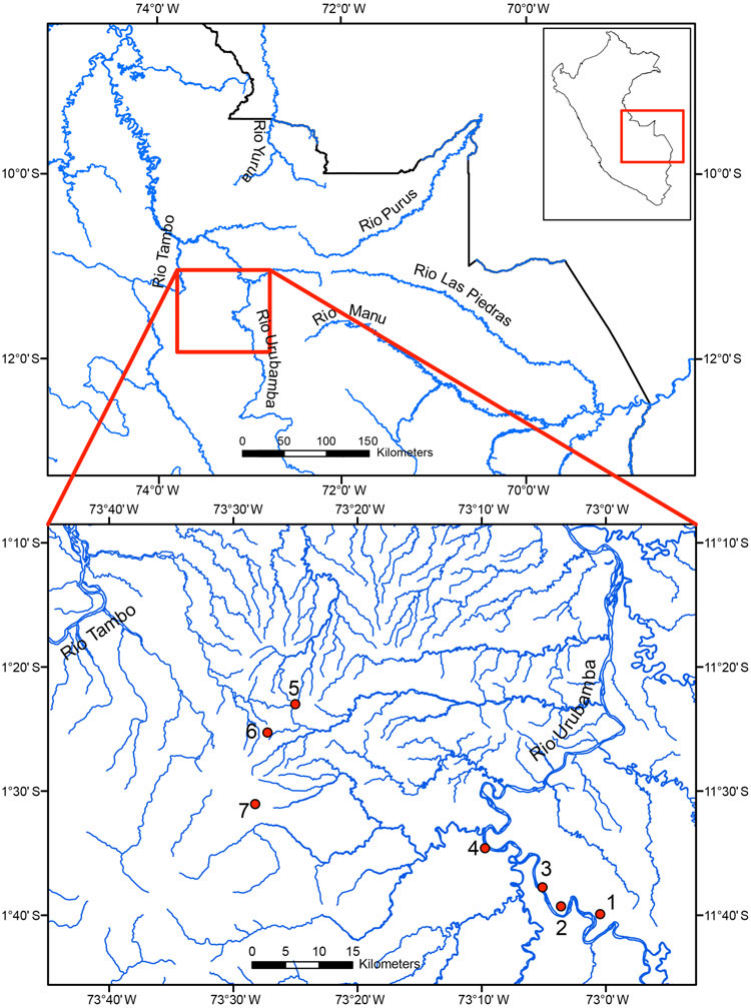
Location of census sites in central–eastern Peruvian Amazonia. Lower Río Urubamba (sites 1–4) and Río Urubamba–Río Tambo interfluvium (sites 5–7)

## Methods

### Study area

The study was conducted on the lower Río Urubamba (four census sites) and in the interfluvium between the Río Urubamba and the Río Tambo (three census sites), located in central–eastern Peru (Fig. 1; Table 1). According to the ecological map of Peru (ONERN 1976), this area corresponds to the tropical humid forest life zone, with physiography varying from lower terraces to sub-montane. At the census sites, forest on hilly and sub-montane terrain dominated, whereas inundable forest was present only in a narrow fringe along some sectors of the lower Río Urubamba.

**Table 1:** Census sites along the lower Rio Urubamba and in the Rio Urubamba–Rio Tambo interfluvium

Area	Census sites^a^	UTM coordinates (easting/northing)	Altitude (m.a.s.l.)	Predominant vegetation type	Degree of human disturbance
Lower Río Urubamba	Pagoreni north (1)	717298/8709604	401	SPFP	High
	Pagoreni west (2)	711711/8714587	374	OPFP	High
	Saniri (3)	708651/8713506	348	OPFP	High
	Mipaya (4)	700406/8719494	418	DPF	High
Río Urubamba–Río Tambo interfluvium	Mapi OX (5)	666848/8726075	759	DPF	Low
	Mashira JX (6)	668648/8736834	594	SPFP	Low
	Kinteroni CX (7)	673425/8741310	680	DPF	Low

*DPF* dense primary forest, *OPFP* open primary forest with “pacales”, *SPFP* semi-dense primary forest with “pacales”

^a^Numbers in parentheses correspond to the points in Fig. 1

Following ERM (2006), we distinguished three principal vegetation types at the census sites:

1. Dense primary forest (DPF): canopy layer between 20 and 25 m with emergent trees >30 m; many trees colonized by epiphytes and lianas; dominant tree species: *Chorisia* sp. (local name: “lupuna”), *Macrolobium angustifolium* (“pashaco”), *Couma macrocarpa* (“leche huayo”), *Cedrelinga cateniformis* (“tornillo”), and *Matisia cordata* (“sacha zapote”); dominant palm species: *Astrocaryum murumuru* (“huicungo”), *Iriartea* sp. (“pona”), and *Phytelephas macrocarpa* (“yarina”).
2. Semi-dense primary forest with “pacales” (SPFP): mixed vegetation with similar proportions of forest characterized by broad-crowned trees and associations of bamboo, *Guadua sarcocarpa* (“pacales”); some trees colonized by epiphytes and lianas; dominant tree species: *M. cordata*, *Dipteris micrantha* (“shihuahuaco”), *Gustavia* sp. (“machimango”), *M. angustifolium*, *Ficus antihelmintica* (“ojé”), *Tetragastris* sp. (“copal”), *Calycophyllum* sp. (“capirona”), and *Inga* spp. (“shimbillo”); dominant palm species: *A. murumuru*, *Socratea* sp. (“cashapona”) and *P. macrocarpa*; parts with extensive bamboo stands very difficult to penetrate, because of the spiny stalks.
3. Open primary forest with “pacales” (OPFP): ca. 80 % of area covered with *G. sarcocarpa*, the remainder consisting of forest patches where *M. angustifolium*, *Tachigalia* sp. (“tangarana”), *Inga* spp., and the palms *Iriartea* sp., *Socratea* sp., and *Euterpe* sp. (“huasaí”) are the most common trees; extremely difficult to penetrate because of spiny bamboo; mean height of the pacales approximately 12 m.

Census sites at Mapi OX, Mashira JX, and Kinteroni CX were undisturbed or slightly disturbed whereas at all other sites trails used for hunting and timber extraction were indicative of much human interference.

### Transect census

At each of the seven sites, five to seven transects 1.5–4.0 km length were opened at least 5 days in advance. Different transect per site were walked in parallel by two teams, each consisting of a researcher and a local field assistant, between 0630 and 1400 hours (diurnal census) and between 1830 and 2200 h (nocturnal census). Transects were walked at a speed of 1.0 km/h, with regular stops every 50 m for 1 min, to carefully screen all forest strata and detect any movement or sound/noise. When a primate group was detected, the perpendicular distance to the transect of the first individual seen, habitat type, and animal activity during detection were recorded. The total length of the diurnal transect walks was 335 km, and 90 km for the nocturnal. The length of transect walks was comparable for the different habitats (Table 2).

**Table 2:** Census time and transect length

Census time	Transect length per vegetation type (km)			Total (km)
	DPF	SPFP	OPFP	
Day	121	111	103	335
Night	28	24	38	90
Total	149	135	141	425

### Data analysis

We calculated number of sightings per 10 km of walked transect. Because of the relatively small number of sightings, we followed Burnham et al. (1980) to calculate group density *D* as *D* = *N* / 2*dL*, where *N* is the number of sightings, *L* the transect length in km, and *d* the mean perpendicular distance to the transect of the first animal detected. For calculation of population densities we multiplied *D* by mean group size, on the basis of complete group counts only. Sightings and density calculations for diurnal species are based on the total 335 km of transect, except for *Ateles chamek*, *Lagothrix cana*, and *Alouatta seniculus*; calculations for these species are based on 232 km only (excluding OPFP), because these large species do not live in this forest type. Density calculation for *Aotus nigriceps* is based on 90 km of transects. Because transect length was very similar for the three habitat types, the proportion of sightings in a given habitat were taken as an indication of relative habitat preferences.

We compared the observed number of sightings per habitat with expected numbers on the basis of transect length by use of a *χ* ^2^ test, performed with Statistica 9.0 (StatSoft 2009). Expected numbers were calculated as Exp = *N*_total_ × transect length_habitat_/transect length_all habitats_, where *N*_total_ is the total number of sightings per species. Because the number of sightings for some species was small, we conservatively set the alpha level at 0.01.

To compare primate communities between habitats, we used group counts per habitat as raw data and calculated similarity coefficients as percentage similarity with Ecological Methodology version 5.2 (© C.J. Krebs 2000). The study adhered to the legal requirements of Peru and the ethical requirements of the ASP.

## Results

One hundred and seventy four groups from nine primate species were encountered during the survey. The most common sightings were of *Callicebus brunneus*, *Saguinus imperator*, and *A. nigriceps*; the least common sightings were of *L. cana* and *A. chamek* (Table 3).

**Table 3:** Group sizes of primates encountered during transect walks

Species (in order of increasing body mass)	Group size		# of groups		Group size range in other areas
	Mean ± SD	Range	Total	With complete group count	
*Saguinus imperator*	4.9 ± 1.1	3–7	41	21	2–5ª
*Saimiri boliviensis*	15.6 ± 3.1	13–19	9	3	30–40^a^
*Aotus nigriceps*	2.8 ± 0.6	2–4	26	18	2–5ª
*Callicebus brunneus*	2.9 ± 0.8	2–4	45	24	2–3ª
*Cebus apella*	8.8 ± 2.4	7–11	14	5	5–10^b^
*Cebus albifrons*	10.3 ± 2.9	6–13	12	6	6–11^b^
*Alouatta seniculus*	5.6 ± 2.1	4–8	14	6	5–8^a^
*Lagothrix cana*	12.6 ± 2.1	11–15	5	3	10–20^a^
*Ateles chamek*	12.5 ± 3	8–17	8	3	2–23^c^

Means are based on counts of complete group counts

^a^Manu National Park: Terborgh and Janson (1983)

^b^Reserva Nacional Pacaya Samiria: Soini (1986)

^c^Sierras de Contamana: Aquino et al. (2005)

Number of sightings and population densities were highest for smaller species and lowest for the largest species (Table 4).

**Table 4:** Sightings and population density estimates in the survey areas

Species	Mean detection distance (m)	Transect length (km)	Number of sightings/10 km transect walk	Population density	
				Groups/km^2^	Individuals/km^2^
*Saguinus imperator*	15	335	1.2	4.1	20.1
*Saimiri boliviensis*	15	335	0.3	0.9	14.0
*Aotus nigriceps*	13	90	2.9	11.1	31.1
*Callicebus brunneus*	17	335	1.3	4.0	11.5
*Cebus apella*	18	335	0.4	1.2	10.6
*Cebus albifrons*	16	335	0.4	1.1	11.3
*Alouatta seniculus*	19	232	0.6	1.6	8.9
*Lagothrix cana*	26	232	0.2	0.4	5.2
*Ateles chamek*	26	232	0.3	0.7	8.3

The smallest groups were observed for *A. nigriceps* and *C. brunneus*, the largest for *Saimiri boliviensis*. Group size ranges were similar to those from other areas in eastern and south-eastern Peru, except for *S. boliviensis* where it was lower in this study (Table 3).

Of the nine species recorded during the survey, eight were seen both in DPF and in SPFP; *L. cana* was only encountered in DPF, and none of the atelines was found in OPFP (Table 5). However, overall the largest number of primate groups was observed in SPFP, but the small and medium-sized species except *A. nigriceps* were also encountered with high frequency in OPFP. *Alouatta seniculus* was more commonly sighted in SPFP (Table 5). For *S. imperator*, *Cebus apella*, and *A. seniculus* observed numbers of sightings per habitat were significantly different from expected numbers on the basis of transect length.

**Table 5:** Proportion (%), number (*N*) of primate sightings according to habitat type, and statistical comparison of observed and expected number of sightings (significant results in bold)

Habitat type	Species^a^ (in order of increasing body mass)									
	S.i.	S.b.	A.n.	C.b.	C.a.	C.al.	A.s.	L.c.	A.c.	Total
Dense primary forest (DPF)										
%	7	–	38.5	4	14	17	29	100	75	19.5
*N*	3	–	10	2	2	2	4	5	6	34
Semi-dense primary forest with “pacales” (SPFP)										
%	44	44	38.5	40	57	41.5	71	–	25	43.1
N	18	4	10	18	8	5	10	–	2	75
Open primary forest with “pacales” (OPFP)										
%	49	56	23	56	29	41.5	–	–	–	37.4
N	20	5	6	25	4	5	–	–	–	65
Total										
%	100	100	100	100	100	100	100	100	100	100
*N*	41	9	26	45	14	12	14	5	8	174
*χ* ^2^ (*df* = 2)	**15.188**	5.400	4.065	**22.148**	4.305	1.986	**10.721**	8.843	5.968	**20.082**
*p*	**<0.001**	ns	ns	**<0.001**	ns	ns	**<0.005**	ns	ns	**<0.001**

^a^S.i., *Saguinus imperator*; S.b., *Saimiri* *boliviensis*; A.n., *Aotus* *nigriceps*; C.b., *Callicebus* *brunneus*; C.a., *Cebus* *apella*; C.al., *Cebus* *albifrons*; A.s., *Alouatta* *seniculus*; L.c., *Lagothrix* *cana*; A.c., *Ateles* *chamek*

Similarity of primate communities was highest between SPFP and OPFP (75 %), intermediate between DPF and SPFP (54 %), and lowest between DPF and OPFP (36 %).

We did not see any phenotypic differences between primate populations of the same primate species on opposite banks of the Río Urubamba. However, the howler monkeys that we encountered during our surveys varied in coat colour between chestnut-red for animals seen at the lower Río Urubamba between 300 and 500 m.a.s.l. and yellowish–golden for those seen in the Río Urubamba–Río Tambo interfluvium above 500 m.

## Discussion

The number of species recorded in our survey (9) is lower than the number (14) reported by Boddiker et al. (1999, 2001) for the lower Río Urubamba. We did not record *Saguinus fuscicollis*, *Saguinus mystax*, *Cebuella pygmaea*, *Pithecia monachus*, or *Cacajao calvus ucayalii*. Boddiker et al. (1999, 2001) also listed potentially occurring species. They considered the presence of *C. pygmaea* and *C. calvus ucayalii* as “not confirmed” for all four of their survey sites, and of the other three species as “confirmed” at one site only (Cashiriari-2 for *S. fuscicollis* and *S. mystax*; Pagoreni for *P. monachus*). However, the presence of *S. mystax* at Cashiriari-2, south of the Río Camisea is highly doubtful:

1. The southern distribution limit of this species has been given by Aquino and Encarnación (1994) as the Río Sheshea and Río Alto Yurua, which was confirmed by recent surveys (G. Gil, personal communication to RA); and
2. Given their ecological similarity, *S. mystax* and *S. imperator* are unlikely to occur sympatrically, which would have to be the case if the former were present at Cashiriari-2. It is possible that *S. mystax* has been mistaken for *S. fuscicollis* or for *S. imperator*.

For *S. fuscicollis* and *P. monachus* we consider the possibility of local absence. Palminteri et al. (2011) demonstrated high variability in the composition of Neotropical primate communities, even between spatially close sites. Furthermore, the closely related *P. irrorata* avoids bamboo forest (Palminteri and Peres 2012) which is present at several of our sites.

Hershkovitz (1987) considered the Río Urubamba as the southern limit of the distribution of *C. calvus ucayalii*. Aquino and Encarnación (1994) agreed with this, but assumed his species had become extinct in the Urubamba region, because Aquino (1988) could not confirm its presence in this area. Instead, Aquino (1988) postulated the Río Sheshea as the southern limit. G. Gil (personal communication to RA) recently reported this species between the Río Sheshea and the Río Alto Yurua. Our survey did not provide evidence of the presence of *C. calvus ucayalii* in the survey area. Because of recent reports of populations of *C. calvus ucayalii* at unexpected sites (Bowler et al. 2009, Vermeer et al. 2011), we would not completely exclude the possibility that this species is present in the Urubamba region, but consider it highly unlikely.

The lack of observation of *C. pygmaea* in our study and the unconfirmed status in the study by Boddicker et al. (2001) suggest the absence of this species from the area.

The different coat colour of howler monkeys at the lower Río Urubamba and in the Río Urubamba–Río Tambo interfluvium supports the notion of Rylands and Brandon-Jones (1999) of high variability of coat colour in *A. seniculus*. We therefore refrain from considering these populations as a separate taxon, *Alouatta juara* (contra Gregorin 2006; Pacheco et al. 2009; see also Voss and Fleck 2011).

Compared with other areas of south-eastern Peru, population densities in this study are lower than those in the Manu National Park (Terborgh and Janson 1983), but higher than those in the Iberia-Iñapari region (Encarnación and Castro 1990). Although methodological differences and total transect length may have an effect, the effect of human intervention and hunting pressure is likely to be more important for these differences. Low population densities in the Iberia–Iñapari region are associated with habitat alteration and destruction and high hunting pressure, since censuses by Encarnación and Castro (1990) were conducted along roads and trails close to human settlements. In contrast, in the Manu National Park rainforests remain unaltered or very little altered, and hunting pressure is absent or very low. In this study, the four census sites along the Río Urubamba were close to Matsiguenga villages, and hunters were encountered during the census walks. This was not the case with the census sites in the Río Urubamba–Río Tambo interfluvium.

Our finding that some species were more commonly sited in SPFP and/or OPFP indicate that in this area of Peruvian Amazonia, forests with bamboo stands are important habitats for primates, particularly for *S. imperator*, *C. brunneus*, and *A. seniculus*, but perhaps also for the three cebid species. For *C. brunneus*, Aquino and Encarnación (1994) had already suggested a preference for “pacales”, but data to support this were lacking. For the moment, we can only speculate on the causes of such preferences. The folivorous *A. seniculus* may benefit from increased leaf quality (higher protein-to-fibre ratio) as a result of increased light exposure in more open forest (Ganzhorn 1995). More open forests are also likely to have a higher abundance of insects (Fowler et al. 1993 compared insect abundance in forest interior vs. edge) which may also be an effect of increased leaf quality (Malcolm 1997). Increased insect abundance might benefit primate species that include insects in their diet, for example *S. imperator*, *C. brunneus*, and the cebids (Terborgh 1983; Heymann and Nadjafzadeh 2013), although Kupsch (2011) did not detect increased prey capture by *Saguinus fuscicollis* in young secondary compared with primary forest.

With the exception of *S. boliviensis*, the range of group size recorded in our study was within or very close to those from other areas in central and southern Peruvian Amazonia. Despite its comparatively small size, these primates are hunted with bow and arrow by the local Matsiguenga.
